# Prevalence and Correlates of Serious Injuries among Adolescents in Mauritius

**DOI:** 10.1155/2021/3733762

**Published:** 2021-12-17

**Authors:** Dickson Okoree Mireku, Jacob Owusu Sarfo, Edward Wilson Ansah, Daniel Apaak, Comfort Armah

**Affiliations:** ^1^Directorate of Academic Planning and Quality Assurance, University of Cape Coast, Cape Coast, Ghana; ^2^Department of Health, Physical Education and Recreation, University of Cape Coast, Cape Coast, Ghana

## Abstract

**Introduction:**

Injuries are a major global health problem that affects teenagers in many countries. Though several studies have been done in many countries, little is known among adolescents in Mauritius. Therefore, our paper explored the prevalence and correlates of serious injuries among adolescents in Mauritius.

**Methods:**

We analysed the 2017 Global School-Based Student Health Survey (GSHS) data from Mauritius, using the Chi-square test and binomial logistic regression analysis with adjusted odds ratio (AOR) at 95% confidence interval (CI).

**Results:**

The prevalence of serious injuries among adolescents in Mauritius stood at 39.0%. Also, the predictors of serious injuries included sex (AOR = 0.70, CI = 0.58–0.81), physical attack (AOR = 0.47, CI = 0.39–0.57), being bullied (AOR = 0.48, CI = 0.48–0.70), suicide ideation (AOR = 0.65, CI = 0.49–0.85), hunger (AOR = 0.65, CI = 0.48–0.86), truancy from school (AOR = 0.77, CI = 0.63–0.93), marijuana use (AOR = 0.54, CI = 0.39–0.76), alcohol consumption (AOR = 0.64, CI = 0.70–0.98), and parental neglect (AOR = 0.83, CI = 0.70–0.98).

**Conclusion:**

The rate of injury among adolescents in Mauritius is moderately high, with sex, suicidal thought, hunger, truancy, drug use, and parental neglect as correlates. There is an urgent need for health promotion interventions at family, community, and school levels to deal with this level of serious injuries and the factors influencing such occurrences among these adolescents in Mauritius.

## 1. Introduction

Globally, injuries are among the top five causes of disability-adjusted life-years among people between 10 and 24 years [1]. Being a victim of childhood injuries has lifelong impacts on education, health, and wellbeing [1–5]. Thus, children exposed to violence and the resulting injuries are more likely to smoke, misuse alcohol and other drugs, engage in other risky behaviours, and are likely to endure a range of physical and mental illnesses later in life [1, 4, 5]. The United Nations Educational Scientific and Cultural Organisation (UNESCO) [6] reported an estimated 200,000 serious injuries and homicides among persons between the ages of 10 and 29. This figure makes the problem of injuries the fourth leading cause of death among young people worldwide.

The prevalence and associated risk factors influencing adolescents' level of serious injuries have been studied across different cultures over the years. In a recent study on serious injuries among adolescents in Ghana, Ackah et al. [7] noted that the prevalence stood at 66%. Other researchers have reported varying prevalence, 21% in Europe [8], 38% in China [9], and 63% in Ethiopia [10]. Furthermore, these studies associated injuries among adolescents with multiple factors like gender, level of education, suicidal behaviours (ideation and attempt), physical fight, fewer close friends, school truancy, marijuana smoking, and the use of amphetamine [7–10]. From the perspective of the island of Mauritius, the 2011 Global School-Based Student Health Survey [11] reported drug usage, including marijuana, 4.8% (male = 7.8%; female = 2.1%), cigarette smoking, 15.1% (male = 7.8%; female = 2.1%), and 74.5% doing so before their 14^th^ birthday, and alcohol, 24.1% (male = 26.4%; female = 21.7%) with as high as 71.2% of the adolescents drinking before age 14 and 29.9% drinking one or more drinks a day. Moreover, the survey indicated that 33.9% of the students engaged in physical fight (male = 47.7%; female = 22.9%), 22.6% experienced physical attack one or more times, 38.8% had serious injuries (male = 47.2%; female = 30.9%) and 34.8 were bullied (male = 41.9%; female = 28.2%). However, this survey failed to account for suicidality and other factors that might influence these variables.

Similarly, the authors found that 15.74% of adolescents in Mauritius had engaged in physical fights [12]. From the study's findings, the physical fight among participants was associated with gender, food deprivation, bullying, cigarette use, and the likelihood of engaging in a physical fight. Clearly, there is a lack of a comprehensive understanding of the interrelations among many psychosocial variables that affect the life and health of adolescents in Mauritius. Since physical fighting is a significant correlate of injuries [7–10], a prevalence above 15% raises some concerns. Furthermore, a ten-year study, 2007 to 2017, among 7,458 adolescents in Mauritius showed a decline in bullying, physical fighting, and passive smoking. Nonetheless, the prevalence of tobacco use was found to increase [13]. Notwithstanding the existing empirical evidence regarding the prevalence and correlates of serious injuries among adolescents, there is a lack of data from island nations like Mauritius.

Taking the paucity of evidence and resultant lack of intervention to prevent and/or reduce the occurrence of such injuries among adolescents, the island of Mauritius will lag in achieving some critical goals and targets in the United Nations (UN) Sustainable Development Goals (SGDs) [14]. There are 17 SDGs and 169 targets supposed to be achieved by the end of the year 2030 [15]. Specific to providing quality education, health, and wellbeing opportunities for its citizens, Mauritius must be concerned about meeting targets 3.5 and 4.1 of SGDs 3 and 4, respectively, by 2030. Goal 3 calls upon nations to “ensure healthy lives and promote wellbeing for all at all ages,” with target 3.5 encouraging all countries to “strengthen the prevention and treatment of substance abuse, including narcotic drug abuse and harmful use of alcohol” (pp. 16). Additionally, Goal 4 entreats UN member countries to “ensure inclusive and equitable quality education and promote lifelong learning opportunities for all.”

Moreover, indicator 4.1 under this goal states that governments should “ensure that all girls and boys complete free, equitable and quality primary and secondary education leading to relevant and effective learning outcomes” (pp. 18). Therefore, understanding serious injuries and providing evidence-based measures to reduce them would make school-going adolescents live healthy lives and increase their wellbeing. This evidence may also create an environment for ensuring quality education, including learning, for all. Therefore, the current study analysed the prevalence and correlates of serious injuries among adolescents in Mauritius using the 2017 Global School-Based Student Health Survey (GSHS).

## 2. Methods and Materials

### 2.1. Research Design

We collated and utilised data from the 2017 GSHS from the Mauritius context [15]. The GSHS is a school-based survey that uses self-administered questionnaires. The survey collects cross-sectional data on the behaviour, health status, and risk factors related to the principal cause of serious injuries and death among adolescents or young adults of school-going age. The World Health Organisation (WHO), with the United States Centres for Disease Control and Prevention and Mauritius' Ministry of Health, conducted the GSHS. With a cross-sectional survey design, data were obtained from member countries of the WHO that have a vital interest in dealing with the general causes of serious injuries and devising preventive measures to avert such occurrences among adolescents of school-going age.

### 2.2. Ethical Consideration Followed in the Study

The questionnaire was piloted before the actual data collection to ensure the reliability and validity of the items. The survey was approved by Middle Tennessee State University Institutional Review Board. The researchers strictly adhered to all ethical considerations and policies from the Ministry of Education (MoE), Mauritius. Subsequently, entry protocol permissions were obtained from MoE, Mauritius, heads of schools, and teachers. Verbal and written agreements were acquired from all the students. For minors, permission was obtained from their parents. Access to the data can be obtained at the Non-Communicable Disease Microdata Repository for WHO [15]; see the link: https://extranet.who.int/ncdsmicrodata/index.php/catalog/669/data-dictionary/F1?file_name=MUBH2017_GSHS_Data_Public_Use.

### 2.3. Sampling

The study participants were adolescents in grades 8–12 of the Mauritius educational system. A two-stage cluster sample design was used to obtain representative information of all the selected schools across the country. During the first stage, schools were selected with probability proportional to enrolment sizes. At the second stage, classes were randomly selected, and all students in the chosen classes qualified to partake in the study. The response rates for the school, students, and overall data were 100%, 84%, and 84%, respectively [15].

### 2.4. Variables

There are two main categories of variables used in the study: the outcome/dependent variable and the explanatory variables. The primary outcome variable was serious injuries detected or reported among the students. It was a defined construct: “whether or not the student was seriously injured one or more times over twelve months before the survey.” The options ranged from zero (0) times to twelve (12) or more times. The present study went further to dichotomise the responses. Those without injuries, i.e., zero injuries, were grouped as “no injury” and given 0 as code, whereas those with at least one or more injuries were coded 1, “serious injuries.” The explanatory variables were categorised as sociodemographic factors (sex, age, and grade), personal attributes (hunger, missed school without permission), drugs and substance use (amphetamine use, current marijuana smoking, and current alcohol use), and psychosocial (number of close friends, physical attacks, suicide {ideation, planning, and attempt}, and bullying).  Table 1 displays the explanatory variables used in the study.

### 2.5. Data Analysis

In all the analyses, the sample weighting method was applied at the school, student, and sex within grade levels to make it representative of the adolescents of the school-going population in Mauritius and minimise bias on various trends nonresponses. Some variables were recorded on a binary scale in this study as in other GSHS studies [7, 16–18]. The current analysis did not include students with ages below 13 and above 17 years since their frequency was below 40 cases. Also, some of the survey items did not apply to these groups [15]. To address the issue of missing data, we used the multiple imputations (MI) technique. The MI technique was used where the missing values exceeded 1%. The missing data ranged from 1% to 16% and were missing at random. We conducted five MI with the automatic imputation method to maintain data quality concerning missing values [7, 19]. Imputed values compared reasonably to observed values and results using the complete case analysis. The final model goodness of fit was checked, and the results revealed no evidence of a lack of fit with our model's attempt to predict serious injuries significantly.

We conducted two stages of primary analysis to measure variables that were strongly associated with serious injuries among the students in the adolescent group in Mauritius. First, we performed bivariate analysis using Pearson Chi-square to estimate the relationship between serious injuries and the explanatory variables. Subsequently, we entered the variables that showed significant association (*p* < 0.05) into a binomial logistic regression model. The results obtained from the analysis were presented with corresponding adjusted odds ratio (AOR) at 95% confidence interval (CI) (*p* < 0.05).

## 3. Results

### 3.1. Background Characteristics of the Adolescents in Mauritius

**Figure 1:**
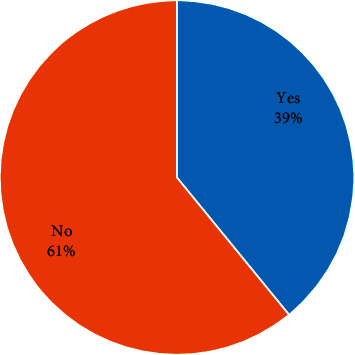
Serious injuries among adolescents attending pretertiary schools in Mauritius.

The overall prevalence of serious injuries in Mauritius adolescents of school-going age was 39% (See Figure 1). Serious injuries among adolescents in Mauritius were associated with several factors. Specifically, a more significant percentage of injury was reported among the male students (45.6%), aged 15–17 (39.5%), and among students in grades eight and nine (42.0%). Also, similar high percentages of injuries were observed among students who went hungry within a period of past 30 days (52.6%), who for 30 days missed school without permission (52.1%), who used amphetamine (61.7%), who smoked marijuana within 30 days to the survey (65.1%), who took drinks containing alcohol (52.7%), and who had no close friends (45.7%). Lastly, we observed a high prevalence of serious injuries in participants who had never experienced physical attacks (59.5%), who had no adequate parental care (45.1%), who had no idea of committing suicide (55.2%), who had a plan of committing suicide (52.2%), who had attempted committing suicide (54.9%), and who were bullied at school (55.2%). See Table 2 for details.

### 3.2. Distribution and Chi-Square Analysis of Serious Injuries across Demographic, Psychological, Personal Attribute Factors, Drugs and Substance Use Factors, and Psychosocial Factors

The Chi-square test conducted showed that students' sex (*χ*^*2*^ = 47.69, *p* < 0.001), students' grade (*χ*^*2*^ = 7.250, *p* < 0.007), hunger condition of students (*χ*^*2*^ = 19.26, *p* < 0.001), truancy (*χ*^*2*^ = 66.50, *p* < 0.001), amphetamine or methamphetamine use (*χ*^*2*^ = 20.85, *p* < 0.001), marijuana smoking (*χ*^*2*^ = 62.66, *p* < 0.001), alcohol use (*χ*^*2*^ = 83.75 *p* < 0.001), having close friends (*χ*^*2*^ = 5.70, *p* < 0.017), having been physically attacked (*χ*^*2*^ = 516.98, *p* < 0.001), having suicide ideation (*χ*^*2*^ = 62.64, *p* < 0.001), having suicide plan (*χ*^*2*^ = 36.71, *p* < 0.001), attempting suicide (*χ*^*2*^ = 46.47, *p* < 0.001), and being bullied (*χ*^*2*^ = 106.44, *p* < 0.001) were significantly associated with serious injuries among adolescents of school-going age in Mauritius.

### 3.3. Logistic Regression Analysis of Significant Factors Associated with Serious Injuries

Table 3 presents the binomial logistic regression analysis results on the factors associated with serious injuries among adolescents of school-going age in Mauritius. The results from the analysis showed that being a male (AOR = 0.689, 95% CI = 0.740–1.025), being physically attacked (AOR = 0.474, 95% CI = 0.393–0.573), being bullied by peers (AOR = 0.581, 95% CI = 0.393–0.573), having suicide ideation (AOR = 0.645, 95% CI = 0.488–0.853), hunger (AOR = 0.646, 95% CI = 0.484–0.862), truancy (AOR = 0.768, 95% CI = 0.633–0.932), marijuana use (AOR = 0.540, 95% CI = 0.385–0.759), alcohol intake (AOR = 0.638, 95% CI = 0.529–0.770), and parental neglect (AOR = 0.827, 95% CI = 0.696–0.981) added significance to the model of serious injury occurrence among adolescents in Mauritius.

## 4. Discussion

The study sought to examine the prevalence and correlates of serious injuries among student adolescents in Mauritius. We found a 39% prevalence of serious injuries among these school-going age adolescents. Moreover, being a male, experiencing physical attacks, being bullied, having suicide ideation, experiencing hunger, playing truancy from school, marijuana and alcohol intake, and parental neglect contributed significantly to severe injuries among the adolescents. Using a nationwide representative sample of adolescents of school-going age between grades 8–12, the overall prevalence of serious injuries found among the adolescents of school-going age in Mauritius was relatively low (39%) as compared with the rates found in Ghana (66.0%) [7] and Liberia (71.6%) [13]. However, this value is higher than the prevalence of injuries recorded in Canada (2.4%) [20]. Nonetheless, the prevalence of serious injuries in Mauritius is similar to that found in China (38%) [9]. This prevalence gives the impression that adolescent behaviours that brought the injuries and related controlling factors in Mauritius need attention.

The current study further showed that demographic factors like sex or gender were significantly associated with serious injuries among these adolescents in Mauritius [7, 9, 13, 20, 21]. From our results, males were more likely to be injured than females. Several studies have reported varied results regarding the role sex differences play in increasing the likelihood of sustaining serious injuries among adolescents. For example, our finding was supported by Jildeh et al.'s [21] observation among school-aged children in Palestine. Accordingly, the study suggested that an increase in male-related injuries was influenced by the nature of sports injuries reported in the study, where the males were likely to play contact and aggressive games [21]. However, our current finding contradicts the recent cross-sectional studies of Ghanaian [7] and Liberian [13] adolescents using GSHS datasets as there was no significant association between sex and serious injuries. The variances in results might have occurred due to the differences in locations, with cultural and socioeconomic variations.

Aside from sex differences, the current study also reported a significant association between serious injuries and personal factors like hunger and truancy among adolescents. The link between hunger and injury behaviours has been observed by Probst et al. [22] across 89 countries. They reported a positive significant dose-response association between hunger and gender on injury due to interpersonal violence. For instance, there was a 30% and 50% increase in male and female odds of injury occurrence, respectively. Therefore, as adolescents get hungry, whether male or female, their interpersonal violence equally rises with related injuries. Similarly, behavioural problems like bully victimisation and physical fights positively correlated with severe injuries among adolescents.

Using the 2010 data from GSHS, it found that about 34% of the adolescent students from Oman also experienced injuries such as fractures and joint dislocation in the preceding year that caused at least a day absence from usual activities or required medical attention [23]. Also, the study reported that 38.4%, 38.8%, and 47.6% of adolescents were bullied at school, physically attacked, and involved in physical fights. Although these aggressive behaviours affected both boys and girls, males were worst affected than females. However, previous evidence among Ghanaian in-school adolescents suggested that the bully resulted from peer victimisation [24]. Such bullying included making fun of the victim with sexual jokes, comments, or gestures that affected both genders, but girls were more likely to have experienced much more than boys [23]. Yet, a more recent study from Portugal [25] revealed that more girls get involved in physical fights than boys, though the current study did not analyse that difference. These negative behaviours among adolescents tend to cause health challenges to them. Furthermore, they could lead to other maladaptive behaviours like using substances including alcohol, tobacco, and other hard drugs and high-level adulthood violence [24].

Evidence from the current findings further indicated that adolescents who experienced minimal parental care, who were bullied at school, wre physically attacked, and conceived suicidal thoughts, were likely to report severe injuries. This finding may have occurred as adolescents' low parental care and psychosocial vulnerability are associated with adverse psychosocial outcomes like suicide [7]. Moreover, the bullied and those physically attacked could retaliate, a situation that can result in fights and attendant injuries. It is essential to recognise that these actions are traumatising and psychologically degrading, leading to suicidal ideation [7], exposing adolescents to these maladaptive behaviours. Therefore, the occurrence of serious injuries can become cyclical until drastic and pragmatic actions are taken to reduce or eliminate the effects of notable explanatory factors. The evidence further suggests that significant correlations exist among explanatory variables in several similar GSHS studies [7, 13, 26]. For example, adolescents who attempted suicide reported relationship problems with family members more often [27] and even abused substances [7]. This finding also calls for more parent-school management collaboration to address factors like the nature of parental care, food intake, bullying behaviours, substance use, physical fight and attacks, suicide ideas, and injuries among adolescents in Mauritius [7–10]. Perhaps, such proactive interventions and policy implementation will help promote the physical and psychosocial health of adolescents in Mauritius.

## 5. Conclusions

The study's findings indicated a moderately high prevalence of serious injuries among adolescents of school-going age in Mauritius. Mauritius' serious injuries prevalence was much higher than Canada, similar to China, but lower than Ghana and Liberia. Also, psychosocial and demographic factors such as hunger, truancy, marijuana smoking, suicide ideation, alcohol use, and parental care were significantly associated with serious injuries among adolescents. We are of the view that, with this rate of serious injuries, the island of Mauritius may not be able to attain the provision of inclusive and equitable quality education and promote lifelong learning opportunities, nor ensure that these school-age adolescents have healthy lives by the year 2030. Hence, there is a need for the government, school authorities, parents, and other stakeholders to strengthen policies and programmes to help shape adolescents' behaviour in schools. Furthermore, finding proactive measures to reduce the prevalence of serious injuries will help Mauritius to achieve some of the targets of the SDGs, particularly SDGs 3.5 and 4.1 (strengthen the prevention and treatment of substance abuse, including narcotic drug abuse and harmful use of alcohol, and ensuring inclusive and equitable quality education and promote lifelong learning opportunities for all).

## Figures and Tables

**Table 1:** Definition of explanatory and measurement coding of variables.

Variable	Survey question	Coding
Sex	What is your sex?	1 = male, 2 = female
Age	How old are you?	1 = 13–14, 2 = 15–17
Grade	In what grade are you?	1 = Grades 8–9; 2 = Grades 10–12
Hunger	Have you gone hungry most of the time or always because there was not enough food at home for the past 30 days?	1 = yes; 2 = no
Close friends	Do you have close friends?	1 = yes; 2 = no
Physical attacks	Have you been attacked physically before?	1 = yes; 2 = no
Suicidal ideation	During the past 12 months, did you ever seriously consider attempting suicide?	1 = yes; 2 = no
Suicidal plan	During the past 12 months, did you make a plan about how you would attempt suicide?	1 = yes; 2 = no
Suicidal attempt	During the past 12 months, did you attempt suicide?	1 = yes; 2 = no
School truancy	During the past 30 days, did you miss classes or school without permission?	1 = yes; 2 = no
Amphetamine use	During your life, did you use amphetamine or methamphetamine (also called ice or yellow)?	1 = yes; 2 = no
Current use of alcohol	During the past 30 days, did you have at least one drink containing alcohol?	1 = yes; 2 = no
Current marijuana smoking	During the past 30 days, did you use marijuana?	1 = yes; 2 = no
Parental care	During the past 30 days, did your parents or guardians understand your problems and worries?	1 = yes; 2 = no

**Table 2:** Bivariate analysis of injuries among adolescents of school-going age in grades eight and twelve in Mauritania (*n* = 2965).

Factors		Serious injury		Chi-square (*χ*^*2*^)	*φ*_*c*_
		Injury (%)	No injury (%)		
*Demographic*					
Sex	Male	642 (45.7%)	766 54.3%	47.69 ^*∗∗∗*^	0.127
	Female	517 (33.2%)	1040 (66.8%)		
Age	13-14	505 (38.6%)	803 (61.4%)	0.23	0.001
	15–17	654 (39.5%)	1003 (60.5%)		
Grade	8-9	503 (42.0%)	694 (58.0%)	7.25 ^*∗∗*^	0.049
	10–12	656 (37.1%)	1112 (62.9%)		

*Personal*					
Hunger	Yes	122 (52.6%)	110 (47.4%)	19.20 ^*∗∗∗*^	0.081
	No	1037 (37.9%)	1696 (62.1%)		
Truancy	Yes	370 (52.1%)	340 (47.9%)	66.50 ^*∗∗∗*^	0.150
	No	789 (35.0%)	1466 (65.0%)		

*Drugs and substance use*					
Amphetamine use	Yes	58 (61.7%)	36 (38.3%)	20.85 ^*∗∗∗*^	0.084
	No	1101 (38.4%)	1770 (61.1%)		
Marijuana smoking	Yes	134 (65.0%)	72 (35.0%)	62.66 ^*∗∗∗*^	0.0145
	No	1025 (37.2%)	1734 (62.8%)		
Alcohol use	Yes	415 (52.7%)	372 (47.268%)	83.75 ^*∗∗∗*^	0.168
	No	744 (34.2%)	1434 (65.84%)		

*Psychosocial*					
Close friends	Yes	128 (45.7%)	152 (54.286%)	5.70 ^*∗*^	0.044
	No	1031 (38.4%)	1654 (61.6%)		
Physically attacked	Yes	409 (59.5%)	278 (40.5%)	516.98 ^*∗∗∗*^	0.230
	No	750 (32.9%)	1528 (67.1%)		
Bullied	Yes	407 (55.1%)	331 (44.9%)	106.44 ^*∗∗∗*^	0.189
	No	752 (33.8%)	1475 (66.2%)		
Parental care	Yes	419 (36.2%)	740 (63.8%)	23.12 ^*∗*^	0.088
	No	814 (45.1%)	992 (54.9%)		
Suicide ideation	Yes	266 (55.2%)	216 (44.8%)	62.64 ^*∗∗∗*^	0.145
	No	893 (36.0%)	1590 (64.0%)		
Suicide plan	Yes	227 (52.2%)	208 (47.8%)	36.71 ^*∗∗∗*^	0.111
	No	932 (36.8%)	1598 (63.2%)		
Suicide attempt	Yes	210 (55.0%)	172 (45.0%)	46.47 ^*∗∗∗*^	0.125
	No	949 (36.7%)	1634 (63.3%)		

Note. ^*∗*^*p* < 0.05,^*∗∗*^*p* < 0.01,^*∗∗∗*^*p* < 0.001.

**Table 3:** Relationship between the significant variables and serious injuries among adolescents.

Variables	B	Wald test (*z*-ratio)	Odds ratio	95% confidence interval for odds ratio	
				Lower	Upper
*Demographic*					
Sex	−.373 ^*∗∗∗*^	19.394	0.689	0.584	0.813
Grade	−0.138	2.771	0.871	0.740	1.025

*Personal*					
Hunger	−0.437 ^*∗∗*^	8.830	0.646	0.484	0.862
Truancy	−0.264 ^*∗∗*^	7.151	0.768	0.633	0.932

*Substance use and abuse*					
Marijuana	−0.616 ^*∗∗∗*^	12.636	0.540	0.385	0.759
Amphetamine	0.052	0.044	1.053	0.648	1.712
Alcohol	−0.449 ^*∗∗∗*^	22.003	0.638	0.529	0.770

*Psychosocial*					
Close friends	−0.032	0.053	0.969	0.739	1.270
Physically attacked	−0.746 ^*∗∗∗*^	59.576	0.474	0.393	0.573
Bullied	−0.543 ^*∗∗∗*^	33.254	0.581	0.483	0.699
Parental care	−0.190 ^*∗*^	4.731	0.827	0.696	0.981
Suicide ideation	−0.438 ^*∗∗*^	9.445	0.645	0.488	0.853
Suicide plan	−0.066	0.214	0.936	0.709	1.237
Suicide attempt	−0.120	0.695	0.887	0.669	1.176
(Constant)	1.437	292.750	4.208		

Note. ^*∗*^*p* < 0.05,^*∗∗*^*p* < 0.01,^*∗∗∗*^*p* < 0.001; Hosmer and Lemeshow test (goodness of fit), *χ*^2^ (8) = 11.890, *p*  = 0.156.

## Data Availability

The data used to support the findings of this study were sourced from the Global School-Based Student Health Survey-Mauritius, 2017, and are available at the Non-Communicable Disease Microdata Repository for the World Health Organization (https://extranet.who.int/ncdsmicrodata/index.php/catalog/669/datadictionary/F1?file_name=MUBH2017_GSHS_Data_Public_Use).

## Additional Points

*Strengths and Limitations of the Study.* We used nationally representative data from Mauritius to investigate the relationship among variables that determine serious injuries among adolescents of school-going age. The study has given good insights into the significant predictors of serious injuries among students within the grades of eight and twelve. The rigorous procedures used in analysing the data have improved the projections of generalising the findings to a similar geographical contest or country where serious injuries must be addressed. Conversely, causal inferences could not be drawn from the results obtained as GSHS uses a cross-sectional survey design. Notwithstanding the limitation, the study provides the “best snapshot” information on severe injuries among adolescents in Mauritius for essential school-based health education interventions by the government, school heads, teachers, and other stakeholders.
